# The linear framework: using graph theory to reveal the algebra and thermodynamics of biomolecular systems

**DOI:** 10.1098/rsfs.2022.0013

**Published:** 2022-06-10

**Authors:** Kee-Myoung Nam, Rosa Martinez-Corral, Jeremy Gunawardena

**Affiliations:** Department of Systems Biology, Harvard Medical School, Boston, MA 02115, USA

**Keywords:** linear framework, graph theory, rational parametrization, Hopfield barrier, Hill functions, post-translational modification systems, gene regulation

## Abstract

The linear framework uses finite, directed graphs with labelled edges to model biomolecular systems. Graph vertices represent biochemical species or molecular states, edges represent reactions or transitions and labels represent rates. The graph yields a linear dynamics for molecular concentrations or state probabilities, with the graph Laplacian as the operator, and the labels encode the nonlinear interactions between system and environment. The labels can be specified by vertices of other graphs or by conservation laws or, when the environment consists of thermodynamic reservoirs, they may be constants. In the latter case, the graphs correspond to infinitesimal generators of Markov processes. The key advantage of the framework has been that steady states are determined as rational algebraic functions of the labels by the Matrix-Tree theorems of graph theory. When the system is at thermodynamic equilibrium, this prescription recovers equilibrium statistical mechanics but it continues to hold for non-equilibrium steady states. The framework goes beyond other graph-based approaches in treating the graph as a mathematical object, for which general theorems can be formulated that accommodate biomolecular complexity. It has been particularly effective at analysing enzyme-catalysed modification systems and input–output responses.

## 1. Introduction

The linear framework is a graph-based approach to biochemical reaction networks [1–3] that arose from studying post-translational modification systems at steady state [4,5]; for reviews, see [6,7]. When it can be deployed, the framework enables a nonlinear biochemical reaction network based on mass-action kinetics to be decomposed into a coupled set of graphs, each of which has a linear dynamics, from which the name ‘linear framework’ is derived. The linear operator is given by the Laplacian matrix of the graph. The main value of the framework has been in giving mathematical access to the steady states of a network as rational algebraic functions of the parameters. This rational algebraic approach has been particularly useful in analysing enzyme-catalysed biochemical networks, such as post-translational modification systems, [4,8–11], and also input–output responses, which arise in several domains, including biochemistry, gene regulation and pharmacology, [7,12–18].

The language of graphs allows essential biological requirements to be specified while leaving other details implicit in the structure of the graph. The framework thereby offers a way to derive general theorems that can rise above the overwhelming molecular complexity that confronts us in modern biology [4,13,16,19]. An example is the rational parametrization theorem for multisite post-translational modification systems, described in §3, which shows that the steady state of such a system is rationally determined just by the enzymes, independently of the number of sites of modification. Despite such successes, the range of applicability of the framework to biochemical reaction networks remains unclear. We will return to this question in the Discussion once we have seen the framework in action.

The case of a single graph coupled to reservoirs of chemical potential corresponds to a continuous-time, finite-state Markov process for which the linear Laplacian dynamics is the master equation [2]. The linear framework thereby offers a graph-based approach to Markov processes. This Markovian interpretation allows thermodynamic concepts like entropy to be specified within the framework, which reduces to equilibrium statistical mechanics for systems at thermodynamic equilibrium. However, importantly, it also provides a setting in which non-equilibrium statistical mechanics is exactly solvable in rational algebraic form (§4). Despite important progress in non-equilibrium physics, the subject remains much less developed than its equilibrium counterpart. The role of energy expenditure in information processing has been particularly elusive and the linear framework offers ways to approach this problem [7,12–14,20–22]. Among the insights to have emerged are that of the ‘Hopfield barrier’ for an information processing task and the identification of the widely used Hill function as the Hopfield barrier for the sharpness of an input–output response [14] (§5).

The mechanisms discussed here provide some of the underpinnings for the study of biological decision-making and time keeping, the subjects of the theme issue in which this paper appears. Post-translational modification systems are found in most biological processes. The pioneering study by Goldbeter and Koshland of a cycle of modification and demodification (§3) provided one of the earliest examples of a molecular switch that can participate in decision-making [23]. The methods described here show how the characteristics of such switches can be understood in terms of the properties of the constituent enzymes (§3). As for input–output responses, they are often convenient ways to summarize the function of mechanisms for which some degree of sharpness is required. Sharpness is often a necessary requirement for the dynamical bistability that may underlie a decision or for the instability that gives rise to a limit cycle oscillation. Hill functions are frequently used to introduce such sharpness in mathematical models (e.g. [24,25]). However, the Hill functions have always lacked rigorous justification, having been originally introduced only as a convenient mathematical family for fitting data [26]. Our results give them, for the first time, a biophysical justification and clarify the conditions under which they can arise biologically (§5).

The sections that follow describe these developments in more detail, drawing in part on recent unpublished work. We follow here the perspective set out in [27], that mathematical models in biology are not descriptions of reality but, rather, accurate descriptions of our (pathetic) assumptions. Hopefully, the assumptions are reasonable in the light of current biological knowledge and of the questions being asked. Having made those assumptions transparent, we try to show how the resulting conclusions are useful for understanding biology. We will also point out some of the open questions and unexplored directions that arise.

The results described here are distributed across papers on a variety of topics, with many of the details buried in supplementary material. We are most grateful to John Tyson and Tim Holt for the opportunity to bring this material together. We hope that what follows will be useful in introducing readers to the capabilities of the linear framework and to some of the open questions that arise from this approach.

## 2. Laplacian dynamics and the Matrix-Tree theorems

**Figure 1.**
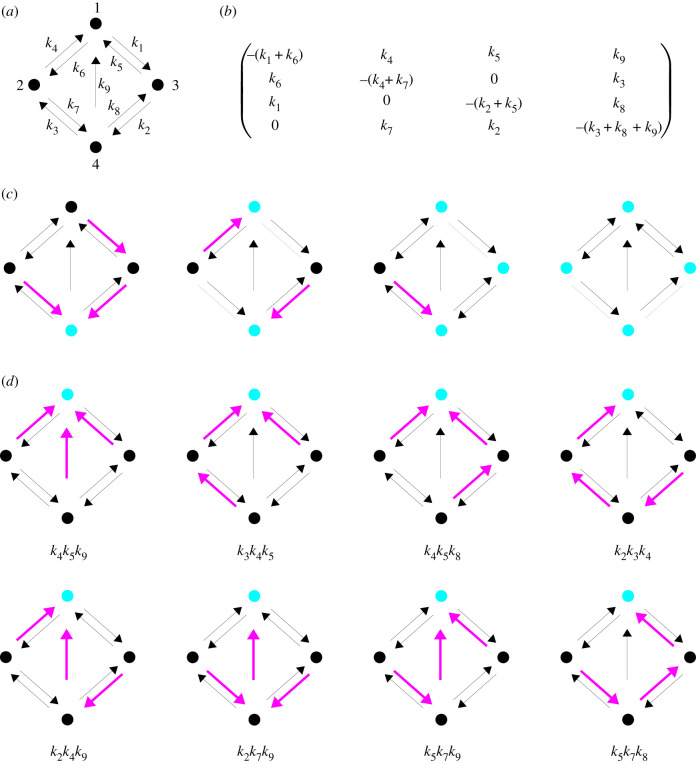
Linear framework graph, Laplacian matrix and spanning trees. (*a*) A strongly connected linear framework graph on four vertices, indexed by 1, …, 4, with symbolic edge labels, *k*_1_, …, *k*_9_. (*b*) The Laplacian matrix corresponding to the graph in (*a*). (*c*) Example spanning forests of the graph in (*a*), demarcated by magenta edges, whose source and target vertices belong to the forest, with root sets (cyan) {4}, {1, 4}, {1, 3, 4} and {1, 2, 3, 4}, reading from left to right. (*d*) All eight spanning trees (magenta edges) rooted at vertex 1 (cyan) for the graph in (*a*), with the corresponding monomial from the MTT in equation (2.4) given below.

We introduce the framework here and outline some of the basic results that provide the foundation for the applications described in subsequent sections. The framework is based on finite, simple, directed graphs with labelled edges. (A simple graph has no more than one directed edge from one vertex to another and has no self loops.) An example graph is shown in figure 1*a*. Let *G* denote a linear framework graph. The vertices of *G* correspond to molecular entities or states and are usually indexed 1, …, *N*, where *N* is the number of vertices. The edges correspond to reactions or transitions and are denoted *i* → *j*. The labels correspond to rates, with dimensions of (time)^−1^, and are denoted ℓ(*i* → *j*). The labels are particularly significant: they can be algebraic expressions which encode the potentially time-dependent interactions between the system described by the graph and its surrounding environment. It is the labels which thereby incorporate the nonlinearity that is usually present. In this section, however, the labels will be treated as abstract parameters, as in *k*_1_, …, *k*_9_ in figure 1*a*. We will explain how labels are interpreted in §3.

A linear framework graph gives rise to a dynamics which is most simply explained as one-dimensional chemistry: each edge is treated as a chemical reaction under mass-action kinetics with the edge label as the rate constant. Since each edge has only one source vertex, the dynamics must be linear and is therefore described by a matrix equation,

2.1 $$\frac{du(t)}{dt}=L(G)\cdot u(t).$$

Here, L(G) is the so-called *Laplacian matrix* of *G*, of size *N* × *N*, and *u*(*t*) is the time-dependent *N* × 1 column vector of vertex ‘concentrations’. These may be actual concentrations if the vertices represent molecular entities or they may be probabilities if the vertices represent molecular states; we will take advantage of both interpretations below. The 4 × 4 Laplacian matrix of the example graph in figure 1*a* is shown in figure 1*b*. Since the chemistry simply moves matter around the graph, without creating or destroying it, there is an obvious conservation law,

2.2 $$u_{1}(t)+\cdots +u_{N}(t)=u_{\mathrm{tot}}.$$

Equation (2.2) corresponds in matrix terms to the column sums of L(G) being zero (figure 1*b*), or $1^{T}\cdot L(G)=0$, where 1 denotes the all-ones column vector and *A*^*T*^ denotes the transpose of matrix *A*. L(G) is a singular matrix and has 0 as an eigenvalue. (We try to keep the notation as light as possible, leaving it to the context to disambiguate the meaning where necessary. If we need to, we will use brackets, as in *u*_tot_(*G*), or a subscript, as in *i* → _*G*_ *j*, to specify which graph is being discussed.) Synthesis and degradation of matter can be accommodated within the graph [1] and some of the consequences are explored in [3].

Laplacian matrices for various kinds of graphs have been widely studied, often with different orientations, signs and scalings [28]. They can be seen from one perspective as discrete approximations to the Laplace–Beltrami operator on a Riemannian manifold [29], so that equation (2.1) resembles a discrete-space diffusion equation. Another interpretation is that equation (2.1) is the master equation of a continuous-time, finite-state Markov process. In this case, the linearity is to be expected, rather than being the consequence of what looks like a rather trivial chemistry. We will explore this stochastic interpretation of equation (2.1) and its thermodynamic implications in §4, after we have looked into the labels in §3.

The framework was introduced to study timescale separation, in which the system under study is assumed to have reached a steady state and one wants to simplify its behaviour accordingly [1]. This is an old technique in physics and it enters biology in the work of Michaelis & Menten [30]. Since equation (2.1) is linear, it is seemingly easy to solve analytically but this is not straightforward to do in terms of the edge labels, for instance by using eigenvalues. Instead, we can exploit graph theory in the form of the *Matrix-Tree Theorems*. First, note that a steady state of equation (2.1), u*, must be in the kernel of the Laplacian, $u^{*}\in \ker L(G)$. If *G* is *strongly connected* then it is not hard to show that this kernel is one dimensional [1],

2.3 dim kerL(G)=1.

Recall that a graph is strongly connected if, given any ordered pair of distinct vertices, (*i*, *j*), there is a path of directed edges from *i* to *j*,

$$i=i_{1}\to i_{2}\to \cdots \to i_{k-1}\to i_{k}=j.$$

The example in figure 1*a* is strongly connected but ceases to be if the edges 1 → 2 and 1 → 3 are removed. Strong connectivity depends only on the *structure* of the graph, which is to say its vertices and edges alone; it is independent of the edge labels. The non-strongly connected case is well understood [2] but we will not need it here.

Despite its simplicity, equation (2.3) is the nub of what follows here. To set up an initial condition, there are as many degrees of freedom for distributing matter among the vertices of the graph as there are vertices. However, the one-dimensional chemistry of the Laplacian dynamics (equation (2.1)) ensures that, when the graph is strongly connected, there is only a single degree of freedom left in the steady state (equation (2.3)). The steady state forgets the initial conditions but remembers the graph. The essential subsequent step is to determine the steady state in terms of the edge labels, which is where the Matrix-Tree Theorems come into play.

The Matrix-Tree Theorems reveal a remarkable property of Laplacian matrices: their minors—the determinants of the square submatrices obtained by removing equal-sized subsets of rows and columns—can be expressed in terms of *spanning forests* of *G*. A spanning forest, *F*, is a subgraph of *G* which contains all vertices in *G* (spanning), has no cycles when edge directions are ignored (forest) and for which each vertex has at most one outgoing edge (which orients the forest). Examples are shown in figure 1*c*. A spanning forest depends only on the structure of a graph. The vertices of *F* with no outgoing edges are called roots. If a forest has only one root, it is a *tree*, so that forests are disjoint unions of trees! Let *ν*(*G*) = {1, …, *N*} denote the vertex set of *G*. If ∅≠U⊆ν(G), let $\Phi_{U}(G)$ be the set of spanning forests of *G* that are rooted at *U*. If *G* is strongly connected, then it is easy to see that $\Phi_{U}(G)\neq \emptyset$. The classical *First-Minors MTT* goes back to Gustav Kirchhoff [31], although the version for labelled directed graphs first appears in Bill Tutte’s PhD thesis [32]; for a statement and proof, see [2]. The *All-Minors MTT* first appeared in the Czech mathematical literature [33] but independent proofs have been given in English (e.g. [34]). These sources give the full statements. We will focus here on using the First-Minors MTT (hereafter, MTT) but the All-Minors MTT comes into its own for some of the new directions mentioned in the Discussion.

Because the first minors give the cofactors of L(G), and hence its adjugate matrix, it is not difficult to see that the MTT leads to a canonical basis element ρ(G)∈kerL(G), which is simply a right eigenvector for the zero eigenvalue. If *H* is any graph, let *λ*(*H*) denote the product of the labels over all the edges in *H*: *λ*(*H*) = ∏_*j*→*k*∈*H*_ℓ(*j* → *k*). The MTT implies that

2.4 $$\rho_{i}(G)=\sum_{T\in \Phi_{\left\{i\right\}}(G)}\lambda (T).$$

The spanning forests in equation (2.4) have only one root and are therefore *spanning trees*. In words, equation (2.4) says that, to obtain a basis element in kerL(G), we take the product of the labels over the edges of each spanning tree rooted at *i* and add these products up over all such trees. This can be done by enumerating all the trees rooted at a vertex, as in figure 1*d*, which becomes the source of the complexity that we will discuss later (§4). The force of the MTT lies in the terms on the right-hand side of equation (2.4) all being positive. Determinants of sub-matrices typically have both positive and negative terms but in the case of Laplacian matrices, there are massive cancellations that result in the sum of positive monomials in equation (2.4). The MTT starkly reveals the underlying positive polynomial dependence on the parameters.

Because of equation (2.3), the steady-state vector, u*(*G*), must be a scalar multiple of *ρ*(*G*), which can be written more concisely as u*(*G*) ∝ *ρ*(*G*). The proportionality constant can be dispensed with by division,

2.5 $$\frac{u_{i}^{*}(G)}{u_{j}^{*}(G)}=\frac{\rho_{i}(G)}{\rho_{j}(G)},$$

for any *i*, *j* ∈ *ν*(*G*), or by normalization to the total,

2.6 $$u_{i}^{*}(G)=\left(\frac{\rho_{i}(G)}{\rho_{1}(G)+\cdots +\rho_{N}(G)}\right)u_{\mathrm{tot}},$$

which exposes the last remaining degree of freedom at steady state in the total concentration of material, *u*_tot_, from equation (2.2).

The positive polynomial dependence arising from the MTT leads to steady-state concentrations being rational algebraic functions of the parameters that are always positive for positive parameter values. We see in equation (2.6) the origin of the rational functions that pervade molecular biology. The Michaelis–Menten and King–Altman formulae in enzyme kinetics [35,36], the Monod–Wyman–Changeux and Koshland–Némethy–Filmer formulae in allostery [37,38] and the Ackers–Johnson–Shea formula in gene regulation [39] can all be derived from equation (2.6) using appropriate graphs [1]. The linear framework not only offers a systematic procedure in place of ad hoc methods, it also reveals the hidden linearity within biochemical reaction networks. (The linearity occurs in the dynamical variables and is captured in equation (2.1); by contrast, the parametric dependence of the dynamics is highly nonlinear, as equation (2.4) shows.) To appreciate how this method works in practice, we need to understand how the edge labels provide an interface between the graph and its environment that brokers linearity out of nonlinear biochemistry.

## 3. Enzyme kinetics and multisite modification systems

**Figure 2.**
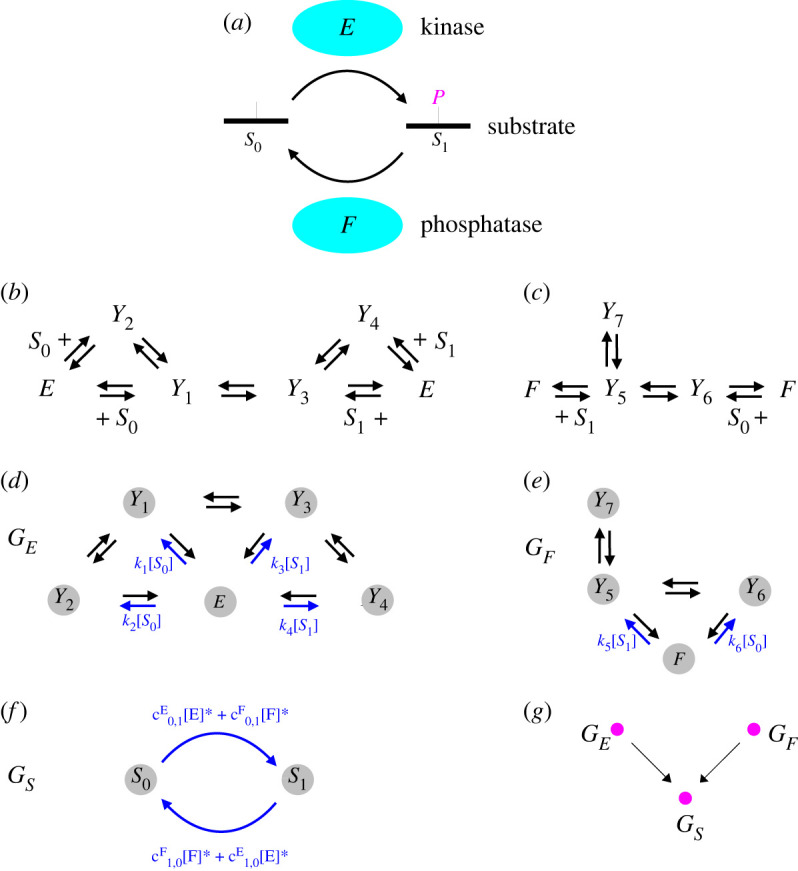
A modification cycle in the linear framework. (*a*) Schematic of a modification–demodification cycle or Goldbeter–Koshland loop with phosphorylation as the modification. (*b*) Hypothetical reaction network for the kinase mechanism in the grammar of equation (3.2). (*c*) Hypothetical reaction network for the phosphatase mechanism with a dead-end complex *Y*_7_, also in the grammar of equation (3.2). (*d*–*f*) Linear framework graphs for the kinase, phosphatase and substrate, respectively (see text). The labels which are simple rates are omitted for clarity; the others, which involve entities from the environment of the graph, are in blue font. (*g*) Metagraph for the modification cycle, where the edges show the labelling relationships between the linear framework graphs represented by the vertices (see text).

Figure 2*a* depicts a modification–demodification cycle, sometimes called a Goldbeter–Koshland loop after the pioneering study that first clarified the switch-like behaviour of this molecular circuit [23]. The modification is shown as a phosphorylation to make it familiar but it could be any small-molecule modification, such as methylation or acetylation. (Ubiquitination and other polypeptide modifications on proteins have a different biochemistry that is not covered here.) The substrate, *S*, could be a protein, so that the process would be one of post-translational modification [40,41], but it could also be some other modifiable molecule such as a carbohydrate or a lipid. *S* is interconverted between its unmodified state, *S*_0_, and its modified state, *S*_1_, by enzymes; for phosphorylation, the forward enzyme, *E*, is a kinase and the reverse enzyme, *F*, is a phosphatase. We will explain here how modification systems like this can be decomposed into linear framework graphs.

We will start by showing how enzymes give rise to graphs which describe their mechanisms. In the literature, it is customary for the mechanisms of *E* and *F* to be described by the Michaelis–Menten reaction scheme,

3.1 $$E+S_{0}⇌ES_{0}\to E+S_{1}.$$

Michaelis and Menten undertook their *in vitro* studies of enzyme kinetics by measuring initial rates of reaction, with essentially no product present [35,42]. They were therefore entirely justified in treating product release as irreversible and adopting equation (3.1). However, equation (3.1) has continued to be used in circumstances, like modification cycles, where there can be substantial amounts of product. *Strongly irreversible* mechanisms like equation (3.1), in which product is unable to rebind to enzyme, are biophysically unrealistic in contexts like this [6,9,43], as Michaelis and Menten well understood [30]. The dangers of assuming strong irreversibility have been repeatedly pointed out [43–45]: it is a limiting assumption that is highly singular [9] and can lead to serious overestimation of the parametric robustness of different dynamical behaviours [11,44]. It is sometimes claimed that equation (3.1) is necessary because it exhibits irreversibility. However, as a matter of thermodynamics, all biochemical transitions are reversible, even if the reverse transition has a relatively much lower rate. Irreversibility is another limiting assumption, which may be appropriate in some physiological contexts. For example, protein kinases and phospho-protein phosphatases appear to operate nearly irreversibly in most cellular contexts. A thermodynamically acceptable way to model this would be to use a *weakly irreversible* mechanism, in which product may rebind but cannot be converted back to substrate. This can be readily accommodated within the linear framework.

The framework allows the use of any enzyme mechanism that can be built up out of the following ‘grammar’ of reactions:

3.2 $$E+S_{*}\to Y_{i},\;Y_{i}\to Y_{j}\;\mathrm{and}\;Y_{k}\to E+S_{*}.$$

Here, $S_{*}$ can be substrate or product and the *Y*’s are arbitrary intermediate enzyme–substrate complexes [9,43]. (There is a mild condition: the linear framework graph for the enzyme mechanism, which we will introduce below, needs to be strongly connected. Reaction networks that do not satisfy this requirement are implausible.) The graph-theoretic machinery described in §2 allows any such mechanism to be described by four aggregated parameters at steady state, as we will see below. This allows us to pay some attention to the details of enzyme mechanisms, which biochemists have worked so hard to disentangle [46–48], rather than persisting with the convenient delusion that all enzymes work the same way as in equation (3.1).

Figure 2*b* shows a hypothetical reaction network for the kinase *E* in the grammar of equation (3.2). In its forward direction, when it converts *S*_0_ to *S*_1_, it has two routes for binding *S*_0_. After the catalytic step between the intermediate complexes *Y*_1_ and *Y*_3_, it releases its product, *S*_1_, also by two routes. The two routes can be thought of as an approximation to what actually happens in a kinase, which has to bind two substrates, *S*_0_ and ATP, in some order and release two products, *S*_1_ and ADP, in some order. Here, ATP and ADP are not explicitly represented as substrates and their effects are assumed to be absorbed into the reaction rates [43]. Enzyme biochemists describe figure 2*b* as a random-order bi-bi mechanism [49]. Phosphatases are much simpler single-substrate hydrolases and a hypothetical mechanism for *F* in the grammar of equation (3.2) is shown in figure 2*c*, in which we have included a dead-end complex. Both mechanisms are fully reversible but they can be made weakly irreversible by removing the reaction from *Y*_3_ to *Y*_1_ for *E* and from *Y*_6_ to *Y*_5_ for *F*.

The approximation in describing the kinase, which is explained in more detail in [43, §2.1], arises because the grammar in equation (3.2) excludes the possibility of a substrate binding to an intermediate complex. This exclusion is important for what follows. It is an interesting question as to whether this constraint can be lifted and a more elaborate grammar developed that would allow multiple-substrate reactions to be more accurately accommodated.

Figure 2*d*,*e* shows the corresponding linear framework graphs *G*_*E*_ and *G*_*F*_ for *E* and *F*, respectively. The vertices are the states of the respective enzyme, namely the free enzyme and whatever intermediate complexes are involved. Vertices have been indexed for convenience by the corresponding enzyme states. The edges correspond to the biochemical reactions in figure 2*b*,*c*. Note that both *G*_*E*_ and *G*_*F*_ are strongly connected. The labels encode how the enzyme states interact with their environment of substrate and product. In particular, an edge label can be an algebraic expression involving concentrations, such as ℓ(*E* → *Y*_2_) = *k*_2_[*S*_0_]. Here, [*X*] denotes the time-dependent concentration of the biochemical entity *X* and *k*_2_ is the second-order rate constant for the bimolecular binding reaction. In this way, the entities that are not represented by vertices, such as *S*_0_ and *S*_1_, are encoded in the edge labels. It is not difficult to see that the linear Laplacian dynamics in equation (2.1) for the graph of any mechanism built up from the grammar in equation (3.2) is just a rewriting of the nonlinear biochemistry of the mechanism, as given by mass-action kinetics [4]. The key requirement here is the *uncoupling condition*: a biochemical entity that appears in an edge label cannot also be represented by a vertex. If this condition is not met, then equation (2.1) would cease to be linear. Uncoupling implies a sharp separation between the vertices and the graph’s environment, for which the labels act as the interface.

Now that we have understood how enzymes can be described in the linear framework, we can turn our attention to the more interesting problem of analysing the modification cycle in figure 2*a* as a whole. We will show that this cycle can be decomposed into three linear framework graphs, two of which are the enzyme graphs that we have already defined. The missing ingredient is a graph for the substrate, *G*_*S*_ (figure 2*f*).

A word on the terminology that we will use from now on: we will write ‘parameters’ or ‘rates’ for rate constants like *k*_2_ in *G*_*E*_ and distinguish them from ‘edge labels’, which may be algebraic expressions involving both parameters and concentrations; ‘aggregated parameters’ are rational algebraic functions of the parameters.

The graphs *G*_*E*_ and *G*_*F*_ are easily derived from the reaction mechanisms in figure 2*b*,*c*, respectively, and correspond to a rewriting of the dynamics; their edge labels involve the time-dependent concentrations of entities in their environment. The situation is more subtle for *G*_*S*_. Here, the vertices are the substrate forms, *S*_0_ and *S*_1_, and the edges represent the action of the enzymes. But what should the labels be? It is hard to imagine labels that can capture the time-dependent dynamics. However, labels can be found which reproduce the steady state. The details go back to [4]; they are described in linear framework terms in [9,43] and summarized in [11].

The edge labels in *G*_*S*_ are defined in terms of aggregated parameters that come from the enzyme mechanisms which implement the edge in question. It is easier to see how these aggregated parameters are defined by stepping back from the particular enzyme examples in figure 2 and working through the construction more generally. Let us consider an arbitrary enzyme *X* that converts between *S*_0_ and *S*_1_ using any mechanism based on equation (3.2) with generic intermediate complexes, *Y*_*i*_. Let *G*_*X*_ be the corresponding enzyme graph, which we assume to be strongly connected. It follows from equation (2.5) that

3.3 $${\left[Y_{i}\right]}^{*}=\left(\frac{\rho_{Y_{i}}(G_{X})}{\rho_{X}(G_{X})}\right){\left[X\right]}^{*},$$

where we have used [−]* to denote concentrations at steady state, so that ${\left[X\right]}^{*}=u_{X}^{*}(G_{X})$. Recalling the MTT prescription in equation (2.4), and following the examples in figure 2*d*,*e*, we see that *ρ*(*G*_*X*_) may involve [*S*_0_]* and [*S*_1_]*. Specifically, any spanning tree rooted at *Y*_*i*_ will have an edge label involving either [*S*_0_]* or [*S*_1_]* but not both, since such labels only occur on outgoing edges from *X*. Hence, $\rho_{Y_{i}}(G_{X})$ is linear in [*S*_0_]* and [*S*_1_]*. By the same token, a spanning tree rooted at *X* has no labels involving either [*S*_0_]* or [*S*_1_]*. Hence, *ρ*_*X*_(*G*_*X*_) is a constant. Accordingly, we can write, at steady state,

3.4 $${\left[Y_{i}\right]}^{*}=(\kappa_{0,Y_{i}}^{X}{\left[S_{0}\right]}^{*}+\kappa_{1,Y_{i}}^{X}{\left[S_{1}\right]}^{*}){\left[X\right]}^{*}.$$

We call the aggregated parameters, $\kappa_{0,Y_{i}}^{X}$ and $\kappa_{1,Y_{i}}^{X}$, *reciprocal generalized Michaelis–Menten constants* (rgMMCs). They tell us through equation (3.4) how much of each substrate is locked up in each intermediate complex at steady state. By equation (2.4), they are given as sums of products of parameters over spanning trees of *G*_*X*_ rooted at *Y*_*i*_. These expressions may be very complicated, reflecting the complexity of *X*’s reaction mechanism, but it is only the aggregate rgMMCs from equation (3.4) that are needed for what follows. The constraints imposed in the grammar of equation (3.2) are essential for the linearity of equation (3.4), from which these aggregated parameters emerge.

We can now define *total generalized catalytic efficiencies* (tgCEs), $c_{0,1}^{X}$ and $c_{1,0}^{X}$, which capture the contribution of the enzyme, *X*, at steady state, to the production rate of *S*_1_ from *S*_0_, which is $c_{0,1}^{X}$, or of *S*_0_ from *S*_1_, which is $c_{1,0}^{X}$. It is easier to describe how this works by returning to the modification cycle in figure 2. For example, for *G*_*E*_ in figure 2*d*,

$$c_{0,1}^{E}=\ell (Y_{3}\to E)\kappa_{0,Y_{3}}^{E}+\ell (Y_{4}\to E)\kappa_{0,Y_{4}}^{E}\;\text{and}\;c_{1,0}^{E}=\ell (Y_{1}\to E)\kappa_{1,Y_{1}}^{E}+\ell (Y_{2}\to E)\kappa_{1,Y_{2}}^{E}.$$

The edge labels on the substrate graph, *G*_*S*_, are then given in terms of these tgCEs and the corresponding steady-state enzyme concentrations, as shown in figure 2*f*. Hopefully, it should now be reasonably clear how this works for any enzyme reaction mechanism. The key point is that, with this labelling, the steady state of *G*_*S*_, as given by equation (2.6), exactly reproduces the steady state of the underlying biochemical reaction network.

We have now decomposed the modification cycle in figure 2 into three linear framework graphs, *G*_*E*_, *G*_*F*_ and *G*_*S*_. This graph decomposition is very useful because it allows us to undertake *variable elimination*: we can use the graphs to iteratively eliminate the steady-state dynamical variables in terms of the free enzyme concentrations, [*E*]* and [*F*]*. The latter can then be determined by the enzyme conservation laws. This elimination procedure works as follows.

First, using the substrate graph, *G*_*S*_, in figure 2*f*, it follows from equation (2.5) that

3.5 $$\frac{{\left[S_{1}\right]}^{*}}{{\left[S_{0}\right]}^{*}}=\frac{\rho_{S_{1}}(G_{S})}{\rho_{S_{0}}(G_{S})}=\frac{c_{0,1}^{E}{\left[E\right]}^{*}+c_{0,1}^{F}{\left[F\right]}^{*}}{c_{1,0}^{F}{\left[F\right]}^{*}+c_{1,0}^{E}{\left[E\right]}^{*}}.$$

Using equation (3.5) together with equation (3.4) in the substrate conservation law at steady state allows [*S*_0_]* and [*S*_1_]* to be expressed as rational functions of [*E*]* and [*F*]*, with *S*_tot_ as an additional parameter. This procedure introduces two further aggregated parameters, the *total rgMMCs* (trgMMCs), $\kappa_{0}^{X}$ and $\kappa_{1}^{X}$:

$$\kappa_{0}^{X}=\sum_{Y_{i}\in \nu (G_{X})}\kappa_{0,Y_{i}}^{X}\;\;\;\text{and}\;\;\;\kappa_{1}^{X}=\sum_{Y_{i}\in \nu (G_{X})}\kappa_{1,Y_{i}}^{X},$$

which determine how much of the substrate is locked up in the intermediate complexes at steady state:

$$\sum_{Y_{i}\in \nu (G_{X})}{\left[Y_{i}\right]}^{*}=(\kappa_{0}^{X}{\left[S_{0}\right]}^{*}+\kappa_{1}^{X}{\left[S_{1}\right]}^{*}){\left[X\right]}^{*}.$$

We can substitute the expressions for [*S*_0_]* and [*S*_1_]* into equation (3.4) to obtain expressions for the [*Y*_*i*_]* as rational functions of [*E*]* and [*F*]*. We have now eliminated all of the variables at steady state in favour of [*E*]* and [*F*]*. Finally, we can substitute the resulting expressions into the conservation laws for the enzymes, to give a pair of simultaneous equations for [*E*]* and [*F*]*,

3.6 $$\begin{array}{cc} & E_{\mathrm{tot}}=R_{E}({\left[E\right]}^{*},{\left[F\right]}^{*})\\ \mathrm{and}\; & F_{\mathrm{tot}}=R_{F}({\left[E\right]}^{*},{\left[F\right]}^{*}). \end{array}\}$$

Here, *R*_*E*_( − , − ) and *R*_*F*_( − , − ) are both polynomials in their two arguments and the tgCEs and trgMMCs become the effective parameters along with *S*_tot_. The modification system in figure 2 has been reduced at steady state to the solution of a pair of simultaneous polynomial equations for the enzyme concentrations (equation (3.6)), with all the other steady-state variables being determined as rational algebraic functions of the enzyme concentrations.

Where does all this machinery get us? First, at the enzyme level, it gives us a set of four aggregated parameters for describing any enzyme mechanism in the grammar in equation (3.2). There is a tgCE and a trgMMC for both the forward and the reverse direction of the enzyme. These parameters determine the nature of the reaction mechanism: when converting *S*_0_ to *S*_1_, *X* is strongly irreversible if, and only if, $\kappa_{1}^{X}=0$ and *X* is weakly irreversible if, and only if, $\kappa_{1}^{X}>0$ and $c_{1,0}^{X}=0$. There is no longer any mathematical reason for persisting with strongly irreversible enzyme mechanisms when they are not appropriate.

Second, and as a consequence of the first point, the Goldbeter–Koshland loop can be analysed without making unreasonable assumptions about the enzyme mechanisms [9,43]. The switch that the loop implements, between *S*_0_ and *S*_1_, cannot be infinitely sharp, as it appears to be when the enzymes are strongly irreversible [23]. Formulae can be given for both the sharpness and the dynamic range of the switch, in terms of the aggregated parameters for arbitrary enzyme mechanisms based on the grammar in equation (3.2) [9]. This brings unexpected insights into the phenomenon of enzyme bifunctionality, in which, for example, a single enzyme acts as both a kinase and a phosphatase. A case in point is the enzyme 6-phosphofructo-2-kinase/fructose-2,6-bisphosphatase, which regulates the switch between glycolysis and gluconeogenesis in the mammalian liver [50]. Why an enzyme should both modify and demodify a substrate has seemed quite mysterious. The methods above show how this architecture enables sharp switching which is also coherent across heterogeneous individual cells, which seems to be particularly relevant for zonation in the liver [9].

Third, and most importantly, the example in figure 2 works in much greater generality for any modification system with multiple sites. Suppose that we have a substrate with any number of sites of modification. Proteins can have an extraordinary number of such sites. The tumour-suppressor p53, which is the protein most frequently mutated in cancers, has over 100 sites of PTM [51]. Even if those sites were all simple binary modifications, which they are far from being, this would imply over 10^30^ possible global patterns of modification, or *modforms*, on a single molecule of p53 [40]. Of course, only a tiny fraction of these modforms can be present at any time but the amount of molecular state that is potentially available is jaw-dropping. (What on Earth is it used for [40]?) Suppose that each of the enzymes responsible for making and unmaking these modifications follows some mechanism based on the grammar in equation (3.2). A given enzyme could have several modforms as substrates and could use a different mechanism on each one. Following the same procedure as described above for the simple modification cycle in figure 2*a*, the multisite modification system can be decomposed at steady state into a collection of enzyme graphs and a substrate graph; the steady-state enzyme concentrations are determined as the solution to a system of simultaneous polynomial equations arising from the enzyme conservation laws, as in equation (3.6); and all the other steady-state dynamical variables are expressed as rational algebraic functions of the enzyme concentrations [4]. The mass-action differential equations describing such a multisite modification system are polynomial and the steady state is therefore the positive part of a real algebraic variety [52]. The main result can be informally stated in the language of algebraic geometry as follows.

**Rational parametrization theorem (RPT)** [4]. *The steady-state variety of such a multisite modification system is birationally equivalent to the variety determined by the conservation laws for the enzymes (equation (3.6)).*

This result was unexpected. Despite the apparent intractability of multisite modification systems, with the number of variables scaling exponentially with the number of sites, the steady-state variety suffers an immense reduction to requiring only the enzyme concentrations to specify it completely, no matter how many sites are present or the complexity of the enzymology. The steady states of all the other variables are given by rational algebraic functions of the steady-state enzyme concentrations and these functions can be explicitly constructed from the graphs. This is not a reduction in the dimension of the variety but, rather, in the dimension of its ambient space. The steady-state variety of a multisite modification system has dimension zero, as does the variety defined by the enzyme conservation laws: both varieties consist of isolated points. The salient issue is that the former variety lives in a potentially extremely high-dimensional ambient space while the latter lives in an ambient space whose dimension is the number of enzymes. It is far more straightforward to deal with the latter than with the former.

The RPT has several consequences. The enzyme conservation laws in equation (3.6) capture the essential nonlinearity of a multisite modification system. These equations can have multiple solutions, which accounts for the multistability that has been found in such systems [5,53]. Everything else in the system is determined as rational functions of these nonlinear solutions. For a system with just two enzymes, equation (3.6) offers ‘pseudo-nullclines’ for determining steady states as the intersections of two curves in $R^{2}$ [5]. By combining the ambient dimension reduction provided by the RPT with homotopy continuation methods for solving polynomial equations, available in software tools like Bertini [54], it becomes possible to map out the ‘parameter geography’ of multistability in multisite modification systems [11]. Several conjectures and problems arise from this, which we lack the space to discuss further here.

The implications of the RPT seem not to have been widely appreciated. It is not unusual to see steady-state varieties of multisite modification systems being algebraically solved by ad hoc methods or, what is worse, being determined by numerical simulation, which requires making specific choices about mechanisms and parameter values rather than exploiting the generality which the RPT offers. The RPT demonstrates how the graph-based approach of the linear framework allows us to rise above some of the molecular complexity within biology, and our lack of knowledge about the details of mechanisms, while still drawing useful conclusions.

The RPT raises several questions that have yet to be explored. For example, although the statement of the RPT captures what has been most useful—that the enzymes alone determine the steady state—it glosses over what happens to the parameters. These are also greatly reduced, from the many original parameters required for the multisite modification system to the few aggregated parameters required for the enzyme equations. Here too, the latter are rational algebraic functions of the former. A more complete statement of the RPT would formalize the relationship between parametrized families of varieties that are rationally related in this way. The language to do this may already exist within algebraic geometry. This may seem an unfamiliar strategy in today’s systems biology but the benefit of such formalization is that it can place the result in a broader mathematical context and clarify the kinds of theorems that we may want to look for elsewhere [55–57].

Further questions arise in asking how far the RPT can be pushed. The possibility was mentioned above of expanding the grammar in equation (3.2) to accommodate reaction mechanisms in which multiple substrates participate. More broadly, does the general form of the RPT, in which a small subset of variables carries the nonlinearity and rationally determines all other variables at steady state, apply to biochemical reaction networks beyond those for single-substrate multisite modification systems?

A potential way to formulate this may be in terms of the ‘metagraph’ determined by the labelling relationship. A preliminary definition could be given as follows. The vertices of the metagraph are linear framework graphs and there is a directed edge from graph *G*_*i*_ to graph *G*_*j*_ if the steady states of *G*_*i*_ enter into the labels of *G*_*j*_. This is a coarse description that takes no account of the details of the labelling, which may need to be specified more carefully, as in [8], but it suffices to pose the problem. The metagraph for the RPT is a star, with the substrate graph at its centre and the enzyme graphs leading to it (figure 2*g*). What happens when the metagraph is more complicated? It seems plausible that, when the metagraph is a tree, a similar result to the RPT might hold, with the reduced set of variables, akin to the ‘enzymes’ in the RPT, corresponding to those metagraph vertices with no incoming edges. When the metagraph is not a tree and has cycles, the situation seems fundamentally different. In this case, the iterative construction of rational functions that is possible in a tree must be replaced by something like the fixed point of a recursive functional equation. Can there still be proper subsets of the variables in terms of which all others are rationally determined? The scope of the metagraph idea has not yet been explored but it seems likely that it could accommodate a broad class of enzyme-catalysed biochemical networks, at least at steady state. If results similar to the RPT could be found in this setting, it would show that biochemical reaction networks have a far richer algebraic structure at steady state than has been apparent up to now.

Mercedes Pérez Millán and Alicia Dickenstein have introduced MESSI (modifications of type enzyme–substrate or swap with intermediates) systems, which substantially generalize the multisite modification systems described here [58], albeit without the graph infrastructure. Other approaches have also been introduced for variable elimination in biochemical reaction networks [59,60]. These developments may provide the tools for exploring the graph-based questions raised above. One of the potential advantages of casting these questions in terms of linear framework graphs is that they can be more readily related to the underlying biochemical architectures that are found in the cell (figure 2) and the aggregated parameters that arise can also be given biochemical meaning. Metatrees of Goldbeter–Koshland loops were introduced in [8], where they were shown to have unique steady states, but they have not yet been placed in the setting of the RPT.

## 4. Markov processes and stochastic thermodynamics

The edge labels in a linear framework graph, *G*, and specifically those which involve entities outside *G*, may be dealt with in different ways. We have seen one of the more elaborate ways in §3, where the entities correspond to vertices in some other graph. A simpler possibility is that they just occupy a buffer, from which they bind and unbind to the system described by *G*. If the ligand *L* is such an entity binding to the vertices of *G*, then the edge labels involving *L* through binding would carry the concentration [*L*] of free ligand. In the absence of synthesis and degradation of *L*, there would be a conservation law for total ligand,

4.1 $$L_{\mathrm{tot}}=[L]+\sum_{i\in \nu (G),L\in i}u_{i}(G),$$

where *L* ∈ *i* denotes those vertices of *G* in which *L* is bound. Equation (4.1), when considered at steady state, would play a similar role to equation (3.6) in determining [*L*]*. If *L* were also bound to other graphs, there would be similar contributions to equation (4.1) from those graphs, and if there were multiple ligands present, a system of simultaneous equations would emerge that is similar to equation (3.6).

A simplification of equation (4.1) is to assume that there is so much ligand present that binding and unbinding to *G* does not change its free concentration, so that [*L*] ≈ *L*_tot_. We call such a buffer a *reservoir*, by analogy with thermodynamic reservoirs such as heat baths. In classical thermodynamics, exchanging heat with a reservoir does not change the reservoir’s temperature. When chemistry is incorporated, exchanging molecular particles with a reservoir does not change its chemical potential, or, in our context, the concentration. If all the entities interacting with a graph are in reservoirs, then their concentrations become constants, like the parameters, which remain unchanged over the timescale of the graph dynamics.

The advantage of this approximation is that the linear framework graph then corresponds to a Markov process [2]. By the latter term, we mean a finite-state, continuous-time, time-homogeneous Markov process, *X*(*t*), specified by a conditional probability distribution, Pr(*X*(*t* + *h*) = *j*|*X*(*t*) = *i*) for *h* ≥ 0, which is independent of *t*. Such a process gives rise to a graph whose vertices are the states of the Markov process and for which there is an edge *i* → *j* if, and only if, the infinitesimal transition rate from *i* to *j* is positive, in which case this rate becomes the edge label,

4.2 $$\ell (i\to j)=\lim_{h\to 0}\frac{\mathrm{Pr}(X(t+h)=j|X(t)=i)}{h}>0.$$

Provided the Markov process is sufficiently well behaved for the infinitesimal rates to exist, linear framework graphs and Markov processes are equivalent. In effect, the graph specifies the infinitesimal generator of the corresponding process. From this perspective, the linear Laplacian dynamics in equation (2.1), with *u*(*t*) now the vector of vertex probabilities and *u*_tot_ = 1 in equation (2.2), becomes the master equation, or Kolmogorov forward equation, for the time evolution of probabilities [2, theorem 4]. We have moved from the deterministic world of concentrations in §3 to the stochastic world of probabilities with the same mathematics.

The graph rarely makes an appearance in the theory of Markov processes, perhaps because the latter has been more concerned with behaviour in the transient regime. (The developments mentioned in the Discussion may be of interest in this respect.) It is also not unusual to see master equations described in a balance of flux form,

$$\frac{du_{i}}{dt}=\sum_{\;j\neq i}(r(i,j)u_{j}-r(j,i)u_{i}),$$

where *r*(*i*, *j*) is what we would call ℓ(*j* → *i*), again without the underlying graph or the Laplacian operator being made explicit. One of the messages of this paper is to point out how useful it can be to take a graph-theoretic approach, even to objects like Markov processes that seem very familiar from a different perspective.

The Markovian interpretation allows us to bring thermodynamics into the picture. For this, we make the standing assumption that whatever graph, *G*, we are dealing with is ‘reversible’, so that if *i* → _*G*_ *j*, then also *j* → _*G*_ *i*. In the interpretation of the graph, it is important that *j* → *i* represents the reverse process to *i* → *j* and not merely some other means to get from *j* to *i* [61]. If i⇋j is any pair of such reversible edges, then the ratio of their edge labels may be given a thermodynamic interpretation in terms of the entropy change during the transition from *i* to *j*,

4.3 $$\frac{\ell (i\to j)}{\ell (j\to i)}=\exp\left(\frac{\Delta S^{\mathrm{env}}+(S_{j}^{\mathrm{sys}}-S_{i}^{\mathrm{sys}})}{k_{B}}\right).$$

Here, Δ*S*^env^ is the total change in the entropy of the environmental reservoirs during the transition *i* → *j*, $S_{i}^{\mathrm{sys}}$ is the internal entropy of vertex *i* and *k*_*B*_ is Boltzmann’s constant. Equation (4.3), which asserts that the label ratio is the exponential of the total entropy change, is known as ‘local detailed balance’, where ‘local’ signifies that this is an assertion about a pair of reversible edges. Equation (4.3) goes back to the pioneering work of Terrell Hill [62] and Jürgen Schnakenberg [63], who began using graph-based descriptions to study biophysical systems. It has now been justified in many different settings [64,65], for example, when the vertices of the Markov process describe the mesoscopic states of some biomolecular system, such as an enzyme or a molecular motor, operating within the cellular environment, with which the system exchanges particles and energy.

Equation (4.3) allows us to interpret the central concept of thermodynamic equilibrium. In mathematics, the word ‘equilibrium’ is often used as a synonym for ‘steady state’. This is not so in physics. Thermodynamic equilibrium is a very special kind of steady state with remarkable properties. In linear framework terms, a graph is at thermodynamic equilibrium if there is no entropy change along any cycle of reversible edges. To formalize this, let $P\;:\;i_{1}⇋i_{2}⇋\cdots ⇋i_{k-1}⇋i_{k}$ be any path of reversible edges in *G* and let *μ*(*P*) denote the product of label ratios along the path,

4.4 $$\mu (P)=\left(\frac{\ell (i_{1}\to i_{2})}{\ell (i_{2}\to i_{1})}\right)\cdots \left(\frac{\ell (i_{k-1}\to i_{k})}{\ell (i_{k}\to i_{k-1})}\right).$$

If *P* is a cycle, so that *i*_1_ = *i*_*k*_, then it follows from equation (4.3) that

$$\mu (P)=\exp\left(\frac{\Delta S^{\mathrm{env}}}{k_{B}}\right),$$

where Δ*S*^env^ is the total entropy change in the reservoirs over one traversal of the cycle. It follows that the graph can be at thermodynamic equilibrium if, and only if, *μ*(*P*) = 1 for all cycles of reversible edges, *P*. This is the ‘cycle condition’ for *G*. It is sometimes known in the reaction network literature as Wegscheider’s condition but its thermodynamic significance was first appreciated by Gilbert Lewis [66]. It says that the product of the edge labels going clockwise around any cycle equals the product going counterclockwise. It is sufficient to check this condition only on the finitely many cycles in any basis of cycles (for details of cycle bases, see [21]).

An equivalent formulation of thermodynamic equilibrium is that it is a steady state, u*, in which any pair of reversible edges, i⇋j, is in flux balance,

4.5 $$u_{i}^{*}\ell (i\to j)=u_{j}^{*}\ell (j\to i),$$

irrespective of any other transitions in which the vertices *i* and *j* are engaged. It is not hard to check that if the cycle condition holds, then any steady state satisfies equation (4.5) and, conversely, if equation (4.5) holds in some steady state, then the cycle condition also holds. Equation (4.5) is ‘detailed balance’ or ‘microscopic reversibility’. It brings out the remarkable nature of thermodynamic equilibrium, in which pairs of reversible transitions become effectively decoupled from each other.

At thermodynamic equilibrium, the prescription for the steady state that comes from the MTT in equation (2.4) can be greatly simplified. Choose any vertex of *G* as a reference, which we take to be 1 by convention. Let *P*_*i*_ be any path of reversible edges from 1 to vertex *i* and let *μ*_*i*_(*G*) = *μ*(*P*_*i*_), which is well defined no matter which *P*_*i*_ is chosen because of the cycle condition. It is not hard to show, again exploiting the cycle condition, that, in vector terms, *μ*(*G*) = *ρ*(*G*)/*ρ*_1_(*G*), where *ρ*(*G*) is the canonical basis element in kerL(G) that comes from the MTT in equation (2.4). Hence, *μ*(*G*) is an alternative canonical basis element for kerL(G). Using equation (2.6), we can write

4.6 $$u_{i}^{*}(G)=\frac{\mu_{i}(G)}{\mu_{1}(G)+\mu_{2}(G)+\cdots +\mu_{N}(G)}.$$

Note that *μ*_1_(*G*) = 1. Using equation (4.3) and a little thermodynamics, the terms *μ*_*i*_(*G*) yield the Boltzmann factors for the vertices, referred to vertex 1 as the zero for the free energy. Equation (4.6) is exactly the prescription for the steady state that comes from equilibrium statistical mechanics, with the denominator being the partition function for the grand canonical ensemble [67]. It follows from equations (4.4) and (4.6) that the only parameters needed at thermodynamic equilibrium are the label ratios, ℓ(*i* → *j*)/ℓ(*j* → *i*), not the individual labels.

**Figure 3.**
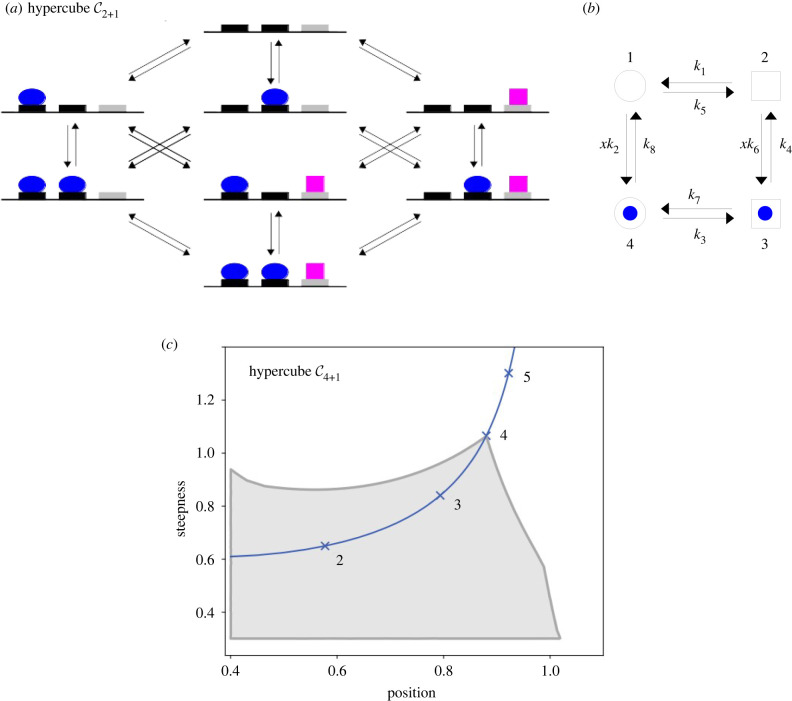
Graphs and position-steepness region. (*a*) Hypercube graph structure $C_{3}$, in the form $C_{2+1}$, with three sites for binding of two ligands, a blue oval to 2 sites and a magenta square to 1 site. One interpretation could be a model of genetic regulated recruitment with the blue oval as a transcription factor and the magenta square as RNA Polymerase. Details of the labels and notation used with such models are given in [7]. (*b*) Linear framework graph to illustrate non-equilibrium complexity, representing a biomolecule in two conformations (circle, square) with a single site for binding of a ligand (blue disc). Vertices are indexed 1, …, 4, parameters are *k*_1_, …, *k*_8_ and *x* is the concentration of ligand. (*c*) The grey region shows the thermodynamic equilibrium (p, s) region for $C_{4+1}$, the regulated recruitment example from (*a*). The input is the concentration of a transcription factor which binds to 4 sites and the output is the steady-state probability of RNA Polymerase bound to the 5th site. The outer curve in darker grey shows the asymptotic boundary of the (p, s) region, for arbitrary parameter values, with the region being truncated to the left and below. The blue curve is the Hill line, the locus of (p, s) points for the Hill functions, with integer Hill coefficients marked.

Linear framework graphs often appear unnecessary when working at thermodynamic equilibrium. This is especially so for physicists. They consider the free energies of vertices to be the fundamental parameters at equilibrium, so that graph edges are irrelevant. What the edges provide, however, are representations of the underlying molecular mechanisms, which are often crucial for interpreting the biology. For example, the widely used hypercube graph structure $C_{n}$ can represent the binding and unbinding of ligands to *n* sites on a biomolecule [14]. Figure 3*a* shows the structure that we denote $C_{2+1}$, reflecting two ligands, one of which binds at 2 sites and the other at 1 site. Hypercube graphs can be used to model a receptor [68], an allosteric system [19] or a gene-regulation system [14]. Such graphs enable *higher-order cooperativities* (HOCs) to be defined at thermodynamic equilibrium, in which binding of a ligand at one site can be modulated by the ligand binding status of multiple other sites [7,14,19]. Prior to this, cooperativity had been largely studied as a pairwise effect involving just two binding sites but there is now compelling evidence that higher-order effects are biologically important [19]. HOCs provide an alternative parametrization of the free-energy landscape and a rigorous language in which to express cooperative interactions involving multiple ligands and sites [7,19]. (HOCs are associated with edges, not vertices, and the cycle condition at thermodynamic equilibrium implies that they are not independent quantities. However, it is straightforward to choose independent subsets of HOCs, in terms of which all other HOCs are rationally expressible [14].) An interesting question is how HOCs arise at a molecular level. It has been known since the pioneering work of Monod, Wyman and Changeux that biomolecular systems with multiple interchanging conformations can create pairwise cooperativity through *allostery* [37,38]. The linear framework can be used to calculate the HOCs that arise from any allosteric conformational ensemble—using the technique of *coarse graining* described in §5—and to prove that such ensembles can implement any pattern of HOCs that is achievable at thermodynamic equilibrium [19].

Despite the algebraic similarity in the formulas for the steady state in equations (4.6) and (2.6), there is a profound distinction between *μ*(*G*) at equilibrium and *ρ*(*G*) away from equilibrium, whose significance becomes clear from the examples calculated below in equations (4.7) and (4.8). At equilibrium, *μ*(*G*) shows that it is only necessary to pick any one path to a given vertex to calculate the steady-state probability of that vertex (up to normalization by the denominator in equation (4.6), which is the partition function). Only the edge label ratios along the path are required. By contrast, away from equilibrium, *ρ*(*G*) shows that every path to the vertex is needed and the MTT in equation (2.4) provides the bookkeeping for this calculation. This poses a major difficulty because the enumeration of spanning trees becomes intractable even for simple graphs. The hypercube graph $C_{2}$ has four spanning trees rooted at each vertex; $C_{3}$ has 384 spanning trees; but $C_{4}$ has 42 467 328 spanning trees [69]! If the combinatorics were not bad enough, *ρ*(*G*) exhibits global parametric dependency: every edge label and parameter in the graph influences the steady-state probability of a vertex. This is startling in its singularity. We can imagine a graph at thermodynamic equilibrium in which the label on a single edge is modified so that the cycle condition is broken. The steady-state probabilities abruptly jump from being locally determined by *μ*(*G*) to being globally determined by *ρ*(*G*), and all parameters, even those arbitrarily far from the edge whose label was changed, now have an effect on the probabilities. The extraordinary path-dependent complexity of calculating non-equilibrium steady states has been a formidable roadblock to progress.

The simple graph in figure 3*b* serves to illustrate the problem. Such a graph, whose structure is the hypercube $C_{2}$, could represent an allosteric system with two conformations, interchanging horizontally, and a single ligand binding site, with binding and unbinding in the vertical transitions. The concentration of the ligand, *x*, appears in the labels for the binding edges. There is a single minimal cycle. Let *f*(*x*) be the steady-state probability of the ligand being bound, considered as a function of *x*; with the notation used in §2, $f\;(x)=u_{3}^{*}(G)+u_{4}^{*}(G)$. Equivalently, for this simple example, *f*(*x*) is also the average number of bound sites at steady state. *f*(*x*) is an input–output function of the kind that we will discuss in more detail in §5. This function looks very different depending on whether the steady state is one of thermodynamic equilibrium or not. If the graph can reach thermodynamic equilibrium, the cycle condition that must be satisfied is

$$k_{1}k_{2}k_{3}k_{4}=k_{5}k_{6}k_{7}k_{8}.$$

With this assumption, we can use equations (4.4) and (4.6) to show that the equilibrium input–output function is of Michaelis–Menten type,

4.7 $$f_{\mathrm{eq}}(x)=\frac{Ax}{B+Ax},$$

where the aggregated parameters are given by the appropriate ratios of rates,

$$A=\left(\frac{k_{2}}{k_{8}}\right)\left(1+\frac{k_{3}}{k_{7}}\right)\;\mathrm{and}\;B=1+\frac{k_{5}}{k_{1}}.$$

If the cycle condition is not satisfied, we can use equations (2.4) and (2.6) to calculate the input–output function. There are four spanning trees rooted at each vertex, which may be enumerated in a similar way to those in figure 1*d*. We find that the non-equilibrium input–output function has degree 2,

4.8 $$f_{\mathrm{ne}}(x)=\frac{A^{*}x+B^{*}x^{2}}{C^{*}+D^{*}x+B^{*}x^{2}},$$

where the aggregated parameters are given in terms of the individual rates,

$$\begin{array}{rl} A^{*} & =k_{1}k_{2}k_{3}+k_{3}k_{5}k_{6}+k_{5}k_{6}k_{8}+k_{1}k_{2}k_{7}+k_{5}k_{6}k_{7}+k_{1}k_{2}k_{4},\\ B^{*} & =k_{2}k_{3}k_{6}+k_{2}k_{6}k_{7},\\ C^{*} & =k_{1}k_{7}k_{8}+k_{1}k_{3}k_{4}+k_{1}k_{4}k_{8}+k_{5}k_{7}k_{8}+k_{3}k_{4}k_{5}+k_{4}k_{5}k_{8}\\ \mathrm{and}\;D^{*} & =A^{*}+k_{2}k_{3}k_{4}+k_{6}k_{7}k_{8}. \end{array}$$

Even for the very simple example in figure 3*b*, the difference between equations (4.7) and (4.8) is striking in both rational structure and parametric complexity.

Physicists have largely dealt with the problem of path-dependent complexity by astute approximations, in which most of the spanning trees are ignored. But this tactic may be particularly problematic in biology in which phenomena often depend on the collective influence of many small effects. Mathematically, the complexity cannot be avoided: equation (2.6) is an exact formula. It is necessary to somehow reorganize the complexity.

Recently, two approaches have emerged which have finally shed some light on this challenging problem. From the mathematical perspective, Pavel Chebotarev and Rafig Agaev have previously interpreted the Faddeev–LeVerrier matrix inversion method for the Laplacian matrix of a graph [70]. Their method allows steady-state probabilities to be recursively and symbolically calculated without explicitly enumerating all the spanning trees. From the physical perspective, non-equilibrium steady-state probabilities can be interpreted as an average of the thermodynamic entropy production along paths [21]. The number of distinct path entropies that are required, and hence the complexity of the average, depends on the *entropy production index* of the graph [21]. If this index is low, calculations can be undertaken away from thermodynamic equilibrium which were previously infeasible [21]. These approaches have provided the first breakthroughs in dealing with path-dependent complexity away from equilibrium.

## 5. Hopfield barriers, coarse graining and Hill functions

The remarkable mathematical difference between the algebraic structure of steady-state probabilities at equilibrium (equation (4.6)) and away from equilibrium (equation (2.6)) strongly suggests that it must have significant biological implications. Surprisingly, these have only slowly emerged. At one level, the physics has always been obvious: we are only at thermodynamic equilibrium when we are dead! The role of energy expenditure in force generation or in pattern formation has been widely studied [71,72]. What has been much less clear, however, has been the implications of energy expenditure for cellular information processing. It was John Hopfield who provided the first insight into this in his pioneering work on kinetic proofreading [73]. The central idea behind his analysis can be restated and updated as follows: *given any information processing task, there is an upper bound to how well it can be undertaken by any mechanism that operates at thermodynamic equilibrium*. We call this bound the *Hopfield barrier* for that task [14]. The only way to exceed this barrier is for the underlying mechanism to operate away from thermodynamic equilibrium.

We have been interested in identifying the Hopfield barriers for various cellular information processing tasks, of which amplification has become the best understood. The problem can be formulated for steady-state input–output responses of biomolecular systems: by how much does the output change for a given change in an input? Here, the system is represented by a linear framework graph, *G*; the input is the concentration, *x*, of some interacting ligand; and the output is any non-negative linear combination of steady-state probabilities,

5.1 $$f\;(x)=\sum_{1\leq i\leq N}\lambda_{i}u_{i}^{*}(G),\;\lambda_{i}\geq 0.$$

Such outputs are often measures of the overall state of the system that are appropriate for the specific biological context, as in the treatment of figure 3*b* in §4. Examples include receptors or allosteric systems responding to ligands, where the output could be the average number of bound sites [19], and gene-regulation systems responding to transcription factors, where the output could be the probability of RNA Polymerase being bound to the gene promoter. The latter kind of input–output response has been widely used to study gene regulation [7], where it represents Mark Ptashne’s concept of regulated recruitment [74]. The hypercube graph $C_{n}$ (figure 3*a*) can model such systems with *n* being the total number of binding sites. The internal complexities of the system are ignored in such a hypercube model and they are considered to exert their effect through the parameter values. The biomolecular system is assumed, under a timescale separation, to have reached a steady state, with changes to the inputs taking place quasi-statically, with sufficiently small and slow increments that the system is not being forced but, rather, relaxes back to steady state after each change. Inputs have typically been examined singly in isolation, although there is no theoretical barrier to greater generality. Such steady-state, single-input, single-output responses have been widely measured and analysed and linear framework graphs are well suited to describing them [14,16,19,75].

The extent to which an input–output response is capable of amplification is usually quantified by some measure of sharpness. The hyperbolic Michaelis–Menten response, *x*/(1 + *x*), has typically been regarded as the baseline, with the Hill functions, H_*h*_(*x*) = *x*^*h*^/(1 + *x*^*h*^), with Hill coefficient, *h* > 0, regarded as displaying sharpness, or ultrasensitivity, which is positive when *h* > 1 and negative when *h* < 1. The Hill coefficient itself is often quoted as a measure of sharpness. The Hill functions can provide surprisingly good empirical fits to input–output response data, going back to their introduction by Archibald Hill to fit the data on oxygen binding to haemoglobin [26]. However, Hill functions, at least those which are not Michaelis–Menten, have no plausible justification [76,77]; they are merely an algebraically convenient functional family for fitting data. That is not the case for the Michaelis–Menten response, for which the linear framework yields theorems that explain its remarkably wide occurrence at the molecular level [16]. The empirical nature of Hill functions has frequently been overlooked, perhaps because they do their job of fitting so well, and they are often used in mathematical models when a sharp response is required without having to justify how the sharpness arises. It is commonplace to see them in models of gene-regulation networks, such as those used to model the transcription–translation circadian clock in metazoa [24]. Given the widespread use of Hill functions in the literature, the justification that we provide for them here may be of some interest.

To analyse sharpness, we use two non-dimensional, intrinsic measures, not just one, which is important for what follows. The definitions build on those previously given in [14] and are taken from the new findings described in [78]. In the generality considered here, input–output functions *f*(*x*) are bounded rational functions for *x* ∈ [0, ∞) and may not be monotonic. As a concrete example, consider the input–output function for a regulated-recruitment model of gene regulation, as described above. If the transcription factor binds at *m* sites, the corresponding graph structure is the hypercube $C_{m+1}$, shown in figure 3*a* for *m* = 2. The remaining site is bound by RNA Polymerase. The input, *x*, is the concentration of the transcription factor and the output is the steady-state probability of RNA Polymerase being bound, which, in figure 3*a*, is the total probability coming from equation (4.6) for the vertices with the magenta square.

We will assume that the output value of *f* is naturally non-dimensional, as it is for the example just described, or that it has been otherwise normalized in a way appropriate to its context. Normalizing the input takes more care because an intrinsic normalization depends on *f*. Let *M*(*f*) and *m*(*f*) be the global maximum and minimum, respectively, of *f*: *M*(*f*) = max_*x*∈[0,∞)_*f*(*x*) and *m*(*f*) = min_*x*∈[0,∞)_*f*(*x*). We normalize the input to *x*_0.5_, which is the minimum positive value for which *f*(*x*_0.5_) is halfway between *m*(*f*) and *M*(*f*):

$$x_{0.5}=\min_{x\in (0,\infty )}\left\{\;x\;|\;\;f\;(x)=\frac{M(f)+m(f)}{2}\right\}.$$

This yields the function *g*(*y*) = *f*(*yx*_0.5_), where *y* is non-dimensional. The input normalization is important for what follows, although the specific definition of *x*_0.5_ does not seem to qualitatively influence the final results. We then define the two measures of sharpness to be the ‘steepness’ of *f*, *s*(*f*), which is the maximum of the absolute value of the derivative of *g*, and the ‘position’ of *f*, *p*(*f*), which is the *y* value at which that maximum is attained,

$$s(f)=\max_{y\in [0,\infty )}\left|\frac{dg}{dy}\right|\;\mathrm{and}\;p(f)=\underset{y\in [0,\infty )}{\mathrm{argmax}}\left|\frac{dg}{dy}\right|.$$

We can now plot *position–steepness regions*, abbreviated to (p, s) regions, assuming that the underlying graphs are at thermodynamic equilibrium, so that the cycle condition holds (§4). Equilibrium parameter values are randomly chosen in some range, the corresponding (p, s) points calculated and the resulting point cloud is incrementally grown until a boundary is reached. The algorithms are elaborated in [14,15,78]. Part of the equilibrium position–steepness region for the regulated-recruitment gene-regulation model described above, based on the hypercube structure $C_{4+1}$, is shown in figure 3*c*.

Three consistent findings emerge from such plots. First, the (p, s) region becomes effectively asymptotically bounded as the parametric range is increased. Some care is needed to state this precisely because the boundary at zero position, *p* = 0, is anomalous: when position becomes very low, steepness can become very high. However, this arises from degenerate functions, as discussed in [14]. The more interesting behaviour occurs away from this boundary. To specify it, choose any *a* > 0, no matter how small. No matter what parameter values are chosen, that part of the (p, s) region within the quadrant [*a*, ∞) × [*a*, ∞) is bounded. Specifically, for all sufficiently large *A* > *a*, the region lies within the finite box [*a*, *A*] × [*a*, *A*]. Figure 3*c* shows the (p, s) region lying within the finite box [0.4, 1.2] × [0.3, 1.2]. The boundedness then leads to the second finding. The (p, s) region exhibits a cusp which lies on the *Hill line*, the locus of (p, s) points for the Hill functions (figure 3*c*). The tip of the cusp is always below the (p, s) point of the Hill function whose coefficient is the number of sites at which the input binds and the cusp approaches closer to this Hill point as the parametric range increases. So, if the input ligand binds at *m* sites of the biomolecular system, the cusp lies below, and asymptotically approaches, the (p, s) of $H_{m}$. Third, if the assumption of thermodynamic equilibrium is dropped, (p, s) points can be found which lie above and to the right of the (p, s) of $H_{m}$.

The asymptotic boundedness of the (p, s) region allows us to draw strong conclusions when experimental data fall outside this region [15]. We can claim that the underlying model is unable to account for the data without having to fit any data. Of particular interest is the possibility that energy expenditure is required to explain the data. This goes to the heart of the striking difference between prokaryotic and eukaryotic gene regulation [7]. The former appears to take place without any expenditure of energy, while the latter uses many sources of energy expenditure, including chromosome and nucleosome reorganization, post-translational modification of histones and co-regulators, and DNA methylation. We believe that this reflects a greatly increased capability for regulatory information processing in eukaryotic genomes. The methods described here provide the theoretical tools for addressing this question [7,13–15].

The three findings described above imply that the Hill function $H_{m}$ is the Hopfield barrier for the sharpness of input–output functions with *m* binding sites for the input ligand. This has been confirmed by numerical plotting of (p, s) regions for several models based on hypercube graphs with different output functions [14,15], including the regulated recruitment model whose (p, s) region is shown in figure 3*c*. However, models could be much more complex than those represented by hypercube graphs and could incorporate many details of the internal mechanisms of the underlying biomolecular system. It seems impossible, on the face of it, to make a definitive statement about the (p, s) regions for all of these models. It turns out, however, that a much stronger statement is possible of the universality of the Hill function as a Hopfield barrier. This relies on the technique of *coarse graining*, which was introduced in [19] to analyse allosteric conformational ensembles.

Suppose *G* is any strongly connected, reversible linear framework graph, which need not satisfy the cycle condition. Given any partition of the vertices of *G*, $\nu (G)=G_{1}\cup \cdots \cup G_{s}$ with $G_{w}\cap G_{z}=\emptyset$ when w≠z, the coarse-grained graph *C*(*G*) is defined as follows. *C*(*G*) has vertices 1, …, *s* corresponding to the subsets of the partition. It has an edge *w* → _*C*(*G*)_ *z* whenever there exists *i* ∈ *G*_*w*_ and *j* ∈ *G*_*z*_ such that *i* → _*G*_ *j*. *C*(*G*) thereby inherits reversibility from *G*. Finally, *C*(*G*) has labels given by

5.2 $$\ell (w\to_{C(G)}z)=Q\left(\sum_{\;j\in G_{z}}\rho_{j}(G)\right).$$

Here, the quantity *Q* is chosen arbitrarily to ensure the dimension of the label is (time)^−1^ but it plays no essential role, as we will see. It is shown in [19] that, with the labelling given by equation (5.2), *C*(*G*) satisfies the cycle condition, even when *G* does not, and, furthermore, that the following coarse-graining equation is satisfied:

5.3 $$u_{w}^{*}(C(G))=\sum_{i\in G_{w}}u_{i}^{*}(G).$$

In other words, the steady-state probability of being at *w* in *C*(*G*) is the same as the steady-state probability of being at any of the vertices of *G*_*w*_ in *G*. This is exactly what we would expect from a coarse graining. Although equation (5.2) is not intuitive, it is the unique specification that allows equation (5.3) to hold, with *C*(*G*) satisfying the cycle condition [19].

It is important to note that the prescription above is not a coarse graining of the dynamics. There is no reason to expect that *C*(*G*) will approximate the dynamics of *G*, only that it reaches the expected steady state under the coarse graining. Because *C*(*G*) satisfies the cycle condition and can therefore reach thermodynamic equilibrium, the only parameters that are required at steady state are the label ratios, so that the quantity *Q* in equation (5.2) cancels out and plays no role.

Coarse graining appears to be a powerful method for making general statements about steady-state behaviour, especially at thermodynamic equilibrium [19], and we expect that it will be broadly useful. To reveal the universality of the sharpness Hopfield barrier, we can consider any strongly connected, reversible graph, *G*, involving the binding of some ligand, *L*, to *m* sites. The graph may involve many other features, such as other ligands, allosteric conformations, co-regulators, patterns of post-translational modifications, scaffold configurations involving locations, and, in the context of gene regulation, chromatin state, nucleosomes, DNA methylation states, etc. (e.g. [12]). No matter how complex the graph, we can always coarse-grain it by bringing together all those vertices of *G* which exhibit the same pattern of binding of *L*. The resulting coarse-grained graph, *C*(*G*), will have the same structure as the hypercube $C_{m}$. (In fact, *C*(*G*) may be a proper subgraph of $C_{m}$ but we will ignore this possibility in the interests of keeping the exposition simple; see [78] for a full discussion.)

For example, the hypercube structure $C_{2+1}$ in figure 3*a*, with the blue ligand as the input, would be coarse-grained into the hypercube structure $C_{2}$ by making the subsets of the partition contain those vertices with the blue ligand bound at exactly the same sites. Since there are two binding sites for the blue ligand, there are four subsets in the partition, each of which contains two vertices, depending on whether or not the magenta ligand is bound.

Of course, the edge labels of *C*(*G*), which arise from equation (5.2), could potentially be very complicated but, nevertheless, we know that *C*(*G*) satisfies the cycle condition with this labelling. What happens to an output function on *G*? It can be shown that, provided *G* itself is at thermodynamic equilibrium, any output function on *G* can be rewritten as a non-negative linear combination of steady-state probabilities of *C*(*G*),

5.4 $$\sum_{i\in \nu (C(G))}\lambda_{i}u_{i}^{*}(C(G)),\;\lambda_{i}\geq 0,$$

where, crucially, the coefficients *λ*_*i*_ do not depend on *x*, as long as *G* is at thermodynamic equilibrium [78]. Comparing with equation (5.1), we see that equation (5.4) defines an input–output function on *C*(*G*), which, as noted above, has the hypercube structure $C_{m}$. It follows that, by varying the coefficients *λ*_*i*_ in equation (5.4) as well as the edge labels imposed on $C_{m}$, while ensuring the cycle condition holds, any input–output function on any graph *G* can be recovered, provided *G* also satisfies the cycle condition. In this way, a universal (p, s) region can be plotted, which looks similar in shape to but is larger in size than that shown in figure 3*c* [78]. In particular, it is bounded in [a, ∞)×[a, ∞) for any *a* > 0 and has the same kind of cusp that falls on the Hill line. Although we have not provided the details here, it should at least be plausible that the following result holds.

**Universal Hopfield barrier for sharpness** [78]. *The Hill function H_*m*_ is the universal Hopfield barrier for the (p, s) sharpness of any thermodynamic equilibrium input–output function with m binding sites for the input ligand, no matter how complicated the underlying Markov process.*

We see that, despite its empirical origin, the Hill function has a deep biophysical justification, as the limit of sharpness that can be achieved at thermodynamic equilibrium. This finally provides a rigorous basis for its widespread use in mathematical models. However, that freedom brings with it a new obligation because we are now in a position to ask what kinds of HOCs are needed to approach H_*m*_. While many different patterns of HOCs can do so, one requirement that seems essential is that HOCs up to the highest possible order are present, with binding being modulated by all other available binding sites; see fig. 9 in [19]. Allostery offers one possible mechanism for implementing such HOCs and allosteric conformational ensembles are now widely seen in all cellular contexts [79]. Further experimental study is needed to understand if this is the principal mechanism that is at work in creating sharp input–output responses.

The universal (p, s) region also provides a surprisingly strong claim when experimental data fall outside the region. No equilibrium Markov-process model, no matter how complicated, can account for such data, thereby offering a powerful incentive to explore non-equilibrium explanations.

The universal Hopfield barrier for sharpness is not yet a theorem, since (p, s) regions have so far only been plotted numerically, but we have no doubt as to its veracity. It is a tantalizing open problem to formulate the appropriate mathematical statement, which requires a better understanding of the shape of the cusp that approaches H_*m*_ asymptotically. The (p, s) region is the image of a map from a space of rational input–output functions to $R^{2}$, which encodes the shape of each function in terms of extrema of its normalized derivative. The shape of the (p, s) region is telling us that the components of this map are highly correlated and constrained in the vicinity of the cusp. We do not yet understand the mathematical form of this relationship.

As mentioned above, if the graph does not satisfy the cycle condition, so that it reaches a non-equilibrium steady state, then it is not difficult to find (p, s) points that lie outside the equilibrium region. The situation away from thermodynamic equilibrium is strikingly different from the universality found at equilibrium: simple families of graphs can be found whose non-equilibrium (p, s) regions cover the entire positive quadrant [80]. Plotting non-equilibrium regions for the hypercube graph structures (figure 3*a*) remains challenging because of the path-dependent complexity described in §4 [14]. Little is known about the shape of these regions, whether or not they also have cusps and, if so, where those cusps lie with respect to the Hill line. The general problem of characterizing non-equilibrium (p, s) regions remains wide open.

## 6. Discussion

Graphs similar to those used here are found elsewhere in biology. The usage that is closest to the linear framework goes back to the non-equilibrium physics of Terrell Hill [62] and Jürgen Schnakenberg [63]. The Matrix-Tree Theorem, specifically the First-Minors MTT which underlies equation (2.4), has been independently rediscovered multiple times in different areas of science, by Hill himself [62], by Raoul Bott when he was working on economics [81], from where it finds its way into quantum field theory [82], and by Mark Freidlin and Alexander Wentzell in their work on random dynamical systems and large deviations [83]; for more of the history, see [2]. Indeed, biochemists should recognize the MTT as the King–Altman procedure in enzyme kinetics [36]. These independent manifestations are usually disconnected from graph theory, where the result had already been established (§2). Many of the statements use their own specialized terms in place of the spanning trees that are fundamental concepts of graph theory. We have tried to avoid such parochial tendencies in the linear framework and to deliberately bring out the connections to areas like graph theory, enzyme kinetics, algebraic geometry, Markov processes and non-equilibrium physics. It is this nexus of overlapping perspectives that gives the framework much of its power and appeal.

The main difference between previous uses of graphs and the linear framework is that the graph is regarded not merely as a picture but as a mathematical object in its own right, in terms of which general theorems can be formulated [4,13,16,19]. Graphs are particularly flexible in allowing certain features, such as a type of structure, or a subgraph, or particular edges or labels, to be specified while leaving other aspects of the graph to vary arbitrarily. A case in point is the grammar of enzyme mechanisms in equation (3.2). This flexibility allows theorems to be proved about processes of interest to biology, such as post-translational modification systems and input–output responses, while accommodating some of the extraordinary complexity and diversity that is present at the molecular level. We can summarize the principal insights from the work described here in the following four points.

— There is a surprising linearity that underlies many biochemical networks, which manifests itself when they can be uncoupled into linear framework graphs (§3). This insight also arises from chemical reaction network theory [1,84] and it hints at a deeper simplicity behind the biochemical complexity that we confront.
— A rational algebraic structure, derived from the Matrix-Tree Theorem in equation (2.4), accompanies the linearity at steady state. The rational algebraic formulae of Michaelis–Menten, King–Altman, Monod–Wyman–Changeux, Koshland–Némethy–Filmer and Ackers–Johnson–Shea, familiar to many biologists, are all instances of equation (2.6) applied to some graph (§2). This gives the quantitative foundation of molecular biology a unity which it has previously lacked. The rationality has been central to the analysis of both post-translational modification systems (§3) and the sharpness of input–output responses (§5).
— There is an equivalence between linear framework graphs, under reservoir assumptions, and finite-state, continuous-time Markov processes (§4). The Laplacian matrix of the graph is the linear operator that defines the master equation. Up to now, this relationship has been exploited at steady state but this is no longer a restriction, as we will mention below. The framework again gives access through equation (2.4) to the rational algebraic structure of steady states, which has not been widely explored within Markov process theory.
— The graph theory also provides an algebraic reformulation of stochastic thermodynamics, not only at equilibrium, where the conventional physics is recovered through a new parametrization based on HOCs, but also, more importantly, away from equilibrium (§4). It thereby enables the analysis of energy dissipation in cellular information processing, from which a new interpretation emerges for that most pervasive, but least justified, of rational functions, the Hill function, as the Hopfield barrier for sharpness (§5).

We noted in the Introduction that the range of applicability of the linear framework remains unclear. As far as Markov processes are concerned, the framework offers an alternative approach based on graph theory (§4). As for biochemical reaction networks, the material in §3 suggests that the framework can be deployed when the metagraph that describes how enzymes act on their substrates is acyclic. We conjectured that a generalized version of the RPT may be true under such circumstances. This restriction rules out systems in which enzymatic action occurs along pathways that feed back, which would yield cyclic metagraphs, but it remains an interesting question whether a recursive structure with fixed points can be uncovered here (§3). The framework is also suited to analysing networks that reach steady states, as opposed to those with more complex dynamics, such as limit cycles.

Finally, two recent developments of the linear framework seem worth mentioning. We had long thought that the rational algebraic approach based on equation (2.4) was limited to the steady state and that the transient regime could only be approached by very different methods. In fact, the linear framework can be extended to the transient regime, at least within the Markov process interpretation (§4). We have shown that the All-Minors MTT (§2) allows first-passage times to be calculated as rational algebraic functions of the parameters [85]. This development comes at an appropriate time in biology, where new experimental methods are giving access to faster dynamics at the molecular scale and raising important questions about the range of validity of steady-state assumptions [86]. Another restriction of the framework, which also seemed unavoidable, has been to finite graphs. However, here too, we have shown that a particular class of semi-infinite ‘cylinder’ graphs can be treated recursively, again exploiting the All-Minors MTT [87]. This development is also timely because it applies, in particular, to integrating models of gene regulation with models of gene expression, for which the number of transcribed mRNA molecules becomes part of the system state [7]. These numbers can now be estimated by single-molecule methods and offer a more informative measure of gene output than the regulated recruitment of RNA Polymerase used in the models described above (figure 3*a*). In the light of these exciting developments, many new questions arise for theoretical exploration, which we hope to report on in subsequent work.

## Data Availability

This article has no additional data.
